# Controllable physicochemical properties of WO*_x_* thin films grown under glancing angle

**DOI:** 10.3762/bjnano.15.31

**Published:** 2024-04-02

**Authors:** Rupam Mandal, Aparajita Mandal, Alapan Dutta, Rengasamy Sivakumar, Sanjeev Kumar Srivastava, Tapobrata Som

**Affiliations:** 1 SUNAG Laboratory, Institute of Physics, Bhubaneswar 751 005, Odisha, India; 2 Homi Bhabha National Institute, Training School Complex, Anushakti Nagar, Mumbai 400 094, India; 3 Department of Physics, Alagappa University, Karaikudi 630 003, India; 4 Department of Physics, Indian Institute of Technology Kharagpur, Kharagpur 721 302, India

**Keywords:** annealing, glancing angle sputter deposition, heterojunction, tungsten oxide, work function

## Abstract

In this work, various physicochemical properties are investigated in nanostructured WO*_x_* thin films prepared by radio-frequency magnetron sputtering for optoelectronic applications. A glancing angle of 87° is employed to grow films of different thicknesses, which are then exposed to post-growth annealing. Detailed local probe analyses in terms of morphology and work function of WO*_x_* films are carried out to investigate thickness-dependent property modulations of the as-deposited and annealed films. The analyses show a reasonably good correlation with photoelectron spectroscopic measurements on the films and the bulk *I*–*V* characteristics acquired on a series of WO*_x_*/p-Si heterojunction diodes. The presence of a critical WO*_x_* thickness is identified to regulate the rectification ratio values at the WO*_x_*/p-Si heterostructures and increase in series resistance within the bulk of the films. The present study provides valuable insights to correlate optical, electrical, and structural properties of WO*_x_* thin films, which will be beneficial for fabricating WO*_x_*-based optoelectronic devices, including photovoltaic cells.

## Introduction

Tungsten oxide (WO*_x_*; *x* ≤ 3) is a popular transition-metal oxide for various optoelectronic devices because of its fascinating optical and electrical properties [1]. WO*_x_* is a wide-bandgap oxide semiconductor with a large excitonic binding energy of 0.15 eV and a high optical absorption coefficient (≥10^4^ cm^−1^ in the UV region) [2]. These, in conjunction with decent carrier mobility (12 cm^2^·V^−1^·s^−1^), make this material an ideal candidate for UV photodetector applications [3]. Because of its octahedral lattice symmetry and partially filled d bands, WO*_x_* is also highly attractive as an electrochromic material for developing modern-day smart windows and display devices [4–7]. Exhibiting various stoichiometric and sub-stoichiometric compositions and polymorphs, WO*_x_* usually behaves as an n-type semiconductor because an unintentional incorporation of a certain amount of reduced W cations is thermodynamically inevitable in these films [8]. It is, therefore, possible to tune the physicochemical properties, such as work function, bandgap, and electrical conductivity, to a large extent by controlling the cationic oxidation state and the film stoichiometry [2]. As a matter of fact, adjustments in the film stoichiometry and microstructure are experimentally viable by the choice of a suitable growth technique [9–11]. As a result, heterostructures having an n-type WO*_x_* layer on various p-type substrates such as p-Si [12–13], Cu_2_O [14], NiO [15], p-ZnO nanowires (NWs) [16], diamond [17], and BiVO_4_ [18], have a great technological importance in the field of heterojunction solar cells, LEDs, and resistance random access memory (RRAM) devices. In this regard, radio frequency (rf) sputter deposition is one of the preferred choices as an industry-compatible method to grow WO*_x_* thin films [2,19–21].

Apart from thin films, nanostructured metal oxides generally possess superior electrochemical properties compared to their bulk counterparts [2]. WO*_x_* nanostructures, exhibiting high chemical and thermal stability, and structural flexibility, have obvious relevance in areas such as photocatalysis [22], electrochromism [23], supercapacitors [24], and lithium batteries [25] and have undergone extensive investigations during the last decades. In this respect, the use of glancing angle deposition (GLAD) to produce high-aspect-ratio nanostructures has certain advantages in terms of a wide range of structural possibilities (such as screws and helical or columnar structures) and reduced complexity (no templates involved) [26]. In addition, film crystallinity and compositional variations in such films can also be adjusted as a function of the growth angle [27–28], making GLAD a promising approach to yield nanostructured (NS) films [29–31]. Electronic devices consisting of multilayers often require information on surface electrochemical property variation of the underlying films induced by structural changes. Therefore, a systematic investigation on the surface work function of the NS-WO*_x_* films as a function of thickness holds the potential for accessing its practical device application.

In this article, we report on tunable structural, optical, and electrical properties of glancing angle-deposited NS-WO*_x_* thin films, where NS-WO*_x_* films of different thicknesses (6–60 nm) are prepared by rf sputtering and exposed to post-growth annealing at 673 K in vacuum (2 × 10^−7^ mbar). The role of increased oxygen vacancy concentration (*O*_V_) on optical bandgap and work function is thoroughly investigated by employing various spectroscopic and microscopic techniques. The systematic investigation of the work function of the films reveals a distinct trend with thickness, originating from the thickness-dependent defect concentration within the films. It is observed that the as-deposited NS-WO*_x_*/p-Si heterostructures are quasi-ohmic in nature. The annealed counterparts exhibit a relatively higher rectification, which points towards a possible defect-dependent Fermi level pinning at the hetero-interface. Overall, our systematic experimental observations demonstrate a wide range of tunability and correlation among several physicochemical properties of glancing angle-deposited WO*_x_* films, which is due to serve as a guide for fabricating WO*_x_*-based optoelectronic devices, including carrier-selective contacts for photovoltaic cells.

## Experimental

NS-WO*_x_* thin films were deposited on ultrasonically cleaned p-Si (100) and soda lime glass substrates of 1 × 1 cm^2^ dimension using a rf magnetron sputtering setup (Excel Instruments). Trichloroethylene, propanol, acetone, and DI water were used to carry out ultrasonication of the substrates for removing organic contaminants. Prior to the deposition, the substrates were properly air-dried. A 99.99% pure WO_3_ target (5 mm thick) was used to grow the WO*_x_* films. The initial pressure in the deposition chamber was 5 × 10^−7^ mbar, and the WO*_x_* films were deposited at 5 × 10^−3^ mbar working pressure by injecting ultrapure Ar gas (99.99%) using a mass flow controller at 30 sccm flow rate. The substrate holder was kept 12.5 cm away from the target at a glancing angle of 87° and 50 W rf power (Advanced Energy) was applied to the target, keeping the substrate holder grounded. Pre-sputtering was carried out for a duration of 1200 s to achieve stability in depositions and contamination-free films. A constant substrate rotation (10 rpm) was maintained to achieve uniform WO*_x_* films. Post-growth annealing of all WO*_x_* films (grown under the same conditions) was performed at 673 K for 60 min in a vacuum environment (3 × 10^−7^ mbar).

The thickness of the films was measured using a surface profilometer (Ambios, XP 200). The surface morphology of the as-deposited and the annealed films was acquired using tapping mode AFM (Asylum Research). AFM images were recorded at different places on each sample to confirm the film uniformity. WSxM software was used to carry out AFM image analysis.

Kelvin probe force microscopy (KPFM) was used to study the local work function of the WO*_x_* films. WO*_x_* samples were removed from the high-vacuum environment right before the KPFM measurements to avoid any contamination in air. For KPFM measurements, a conductive tip (Ti/Pt coated) having a resonance frequency of approx. 70 kHz, a stiffness of approx. 2 N·m^−1^, and a radius of curvature of approx. 30 nm was used for KPFM measurements. To examine the uniformity in work function values of each film, different regions on the sample surface were mapped. Also, variable scan angles and scan speeds (0.2 to 1 Hz) were used to avoid the undesired presence of artefacts in the measured data. A dry nitrogen atmosphere ensured a low humidity level of 8–10% during all the KPFM measurements [29]. Further, the *I*–*V* measurements on the WO*_x_*/p-Si heterostructures were performed by preparing Ag electrodes (1 mm diameter) on top of the WO*_x_* films and p-Si substrates. The *I*–*V* data were acquired by applying DC voltage sweeps (−1 V→0 V→+1 V) using a Keithley source meter (Model 2400, Tektronix, USA) in a sandwiched configuration. During the measurements, the bias voltage was applied to the p-Si substrates, whereas the WO*_x_* films were kept grounded.

The crystallinity of the WO*_x_* films was examined using X-ray diffraction (XRD) (Bruker) under Bragg–Brentano geometry (θ–2θ) in an angular window of 2θ = 20° to 80°. The chemical composition of the WO*_x_* films was identified using X-ray photoelectron spectroscopy (XPS) measurements (PHI 5000 VersaProbeII, ULVAC – PHI, INC) with a monochromatic Al Kα source (*h*ν = 1486.6 eV), and a microfocus (100 µm, 15 kV, 25 W) arrangement along with a multichannel detector and a hemispherical analyser. The microstructure of the WO*_x_* films was studied in cross-section view mode using a field-emission scanning electron microscope (FESEM) (Carl Zeiss). The samples were cleaved using a diamond cutter and placed on the SEM sample holder with the cross-sectional area facing the electron beam. All SEM images were captured using 5 keV electrons under the InLens configuration. The optical characteristics of the films were examined using a UV–Vis–NIR spectrophotometer (Shimadzu-3101PC) equipped with an unpolarised light source (300–1200 nm wavelength range).

## Results and Discussion

**Figure 1:**
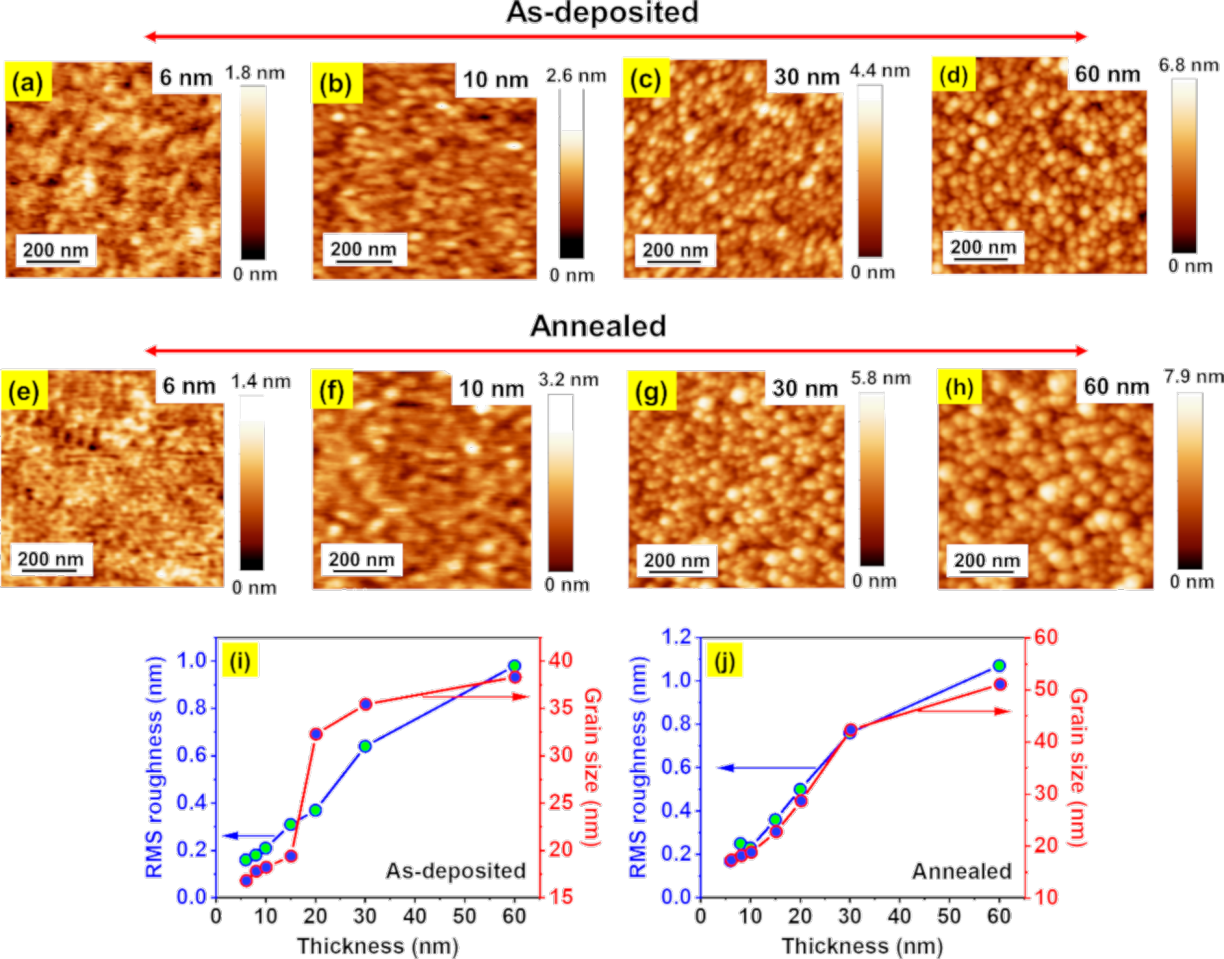
AFM height images of (a–d) as-deposited and (e–h) vacuum-annealed WO*_x_* films grown at a fixed glancing angle of 87°. (i, j) Variations in RMS roughness and grain size with WO*_x_* films thickness before and after annealing, respectively.

Figure 1a–d depicts AFM topographic images of as-deposited WO*_x_* films having thicknesses of 6, 10, 30, and 60 nm on p-Si substrates. It is observed that the films are granular in nature. RMS roughness (blue-green circles) and average grain size (red-blue circles) increase as the film thickness increases from 6 to 60 nm (Figure 1i). Figure 1e–h shows the AFM images of vacuum-annealed (at 673 K for 1 h) WO*_x_* films prepared by the same set of deposition conditions. Similar to the as-deposited ones, all annealed WO*_x_* films possess prominent granular structures and an increasing trend in grain size and RMS roughness with film thickness (Figure 1j). However, the annealed films show bigger grain sizes compared to the respective as-deposited ones. It is noted that the WO*_x_* films deposited on glass substrates show similar trends in grain sizes with film thickness (data not shown here). Additionally, cross-sectional SEM images of 60 and 120 nm thick films are depicted in Figure S1a and Figure S1b (Supporting Information File 1), respectively, confirming the compact growth of the columnar nanostructures originating from the GLAD geometry [32]. Here, the higher thickness is presented only to demonstrate the columnar nanostructure formation in a clearer way. Based on the AFM and SEM analysis, henceforth, the films will be referred to as NS-WO*_x_* films.

**Figure 2:**
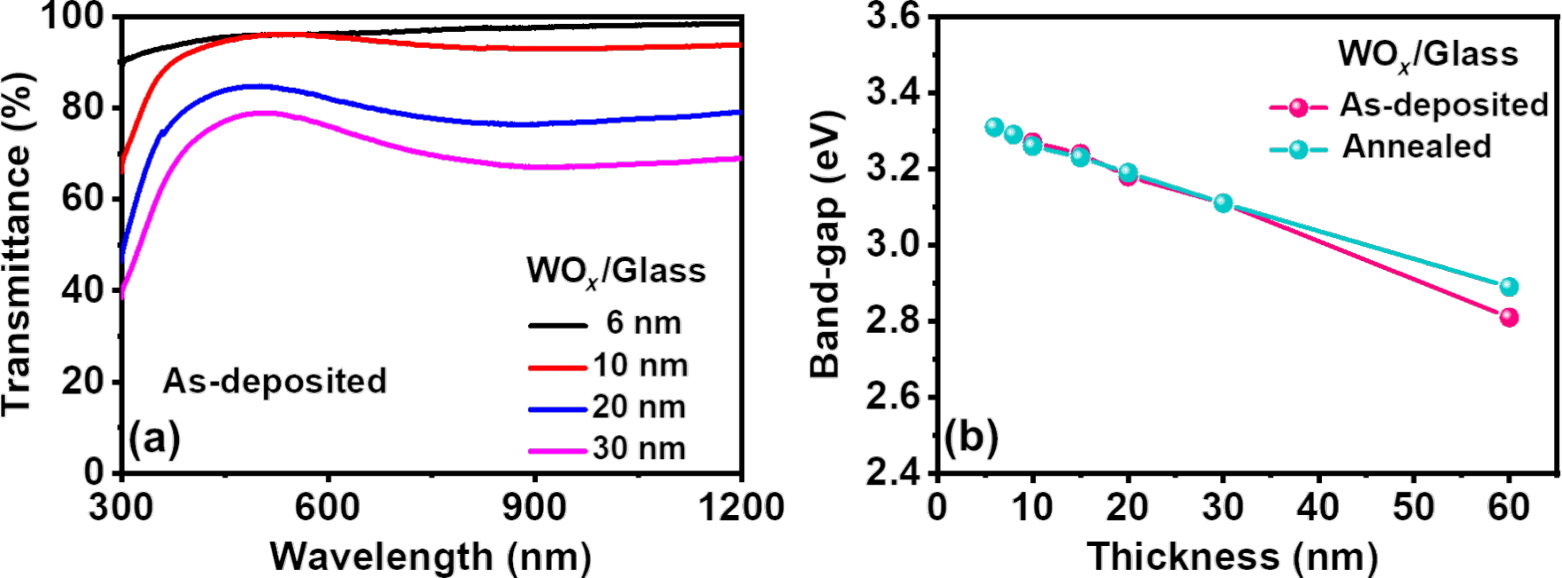
(a) Transmittance plots of the as-deposited NS-WO*_x_* films and (b) bandgap variation with film thickness for as-deposited and annealed films.

Regarding the optical properties, the transmittance spectra of the as-deposited NS-WO*_x_* films on glass show the highest transmittance (more than 90%) over the spectral range of 300 to 1200 nm for 6 nm films, which reduces to 78.4% for the 30 nm films (Figure 2a). The absorption edge, having a sharp drop in the UV region, shifts towards higher wavelengths with increasing thickness values [33]. The shift of the absorption edge to higher wavelengths is likely to be associated with the bandgap (*E*_g_) variation in the NS-WO*_x_* films. The value of *E*_g_ is estimated by employing the well-known Tauc’s equation [34]:


[1] $$\alpha =k{\left(h\nu -E_{\text{g}}\right)}^{n}/h\nu ,$$

where *h*v is the energy of the incident photons (in eV), α is the optical absorption coefficient, *k* is a constant, and *n* is a constant whose value depends on the type of transition (*n* = 2 for direct and *n* = 1/2 for indirect transitions). The optical bandgap values (considering an indirect transition in WO_3_) of the as-deposited and annealed films are estimated from (α*h*v)^1/2^ versus *h*v plots (Tauc plots, see Figure S3 in Supporting Information File 1).

Figure 2b shows the optical bandgap variation of WO*_x_* films before and after annealing as a function of thickness, wherein the bandgap decreases for increasing film thickness (from 6 to 60 nm) in both cases [35]. The estimated bandgap range (2.81–3.31 eV) in various NS-WO*_x_* films is in line with the literature [36–37].

**Figure 3:**
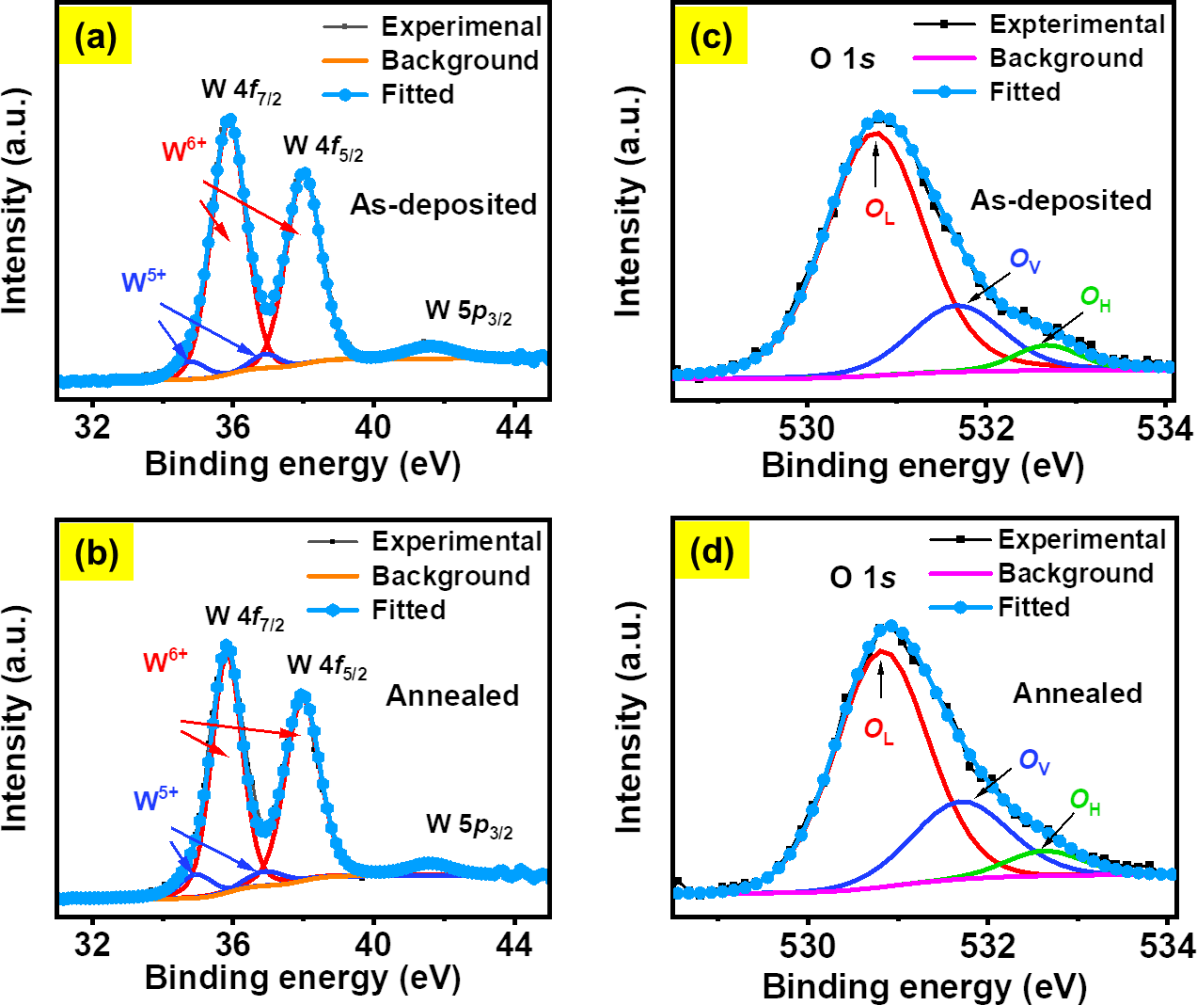
(a, c) XPS core-level spectra for W 4f and O 1s, respectively, of a 6 nm as-deposited WO*_x_* film. (b, d) W 4f and O 1s spectra of the film after vacuum annealing.

In order to understand the observed variability in bandgap energies, we recall that the optical bandgap of this class of materials is a function of defect density and stoichiometric composition, which is mainly governed by the *O*_V_ concentration within the films [39–40]. To probe any possible variation in *O*_V_ concentration and stoichiometry, chemical analysis on the NS-WO*_x_* films is conducted using XPS measurements. Figure 3a–d depicts the XPS core-level spectra of W 4f and O 1s for as-deposited and annealed films, each having a thickness of 6 nm. The W 4f spectra are deconvoluted into two major and two minor peaks using Gaussian–Lorentzian curve fitting after Shirley background subtraction [38]. The two major symmetric peaks at 35.87 and 38.00 eV binding energies correspond to the 4f_7/2_ and 4f_5/2_ levels (spin–orbit splitting: 2.13 eV), respectively, indicating the presence of W^6+^ in the as-deposited WO*_x_* films [38–39]. The two minor peaks at 34.78 and 36.92 eV can be designated to 4f_7/2_ and 4f_5/2_ levels of the W^5+^ oxidation state [40]. Similarly, the presence of W^6+^ and W^5+^ is observed in the annealed WO*_x_* films, where the W 4f_7/2_ and W 4f_5/2_ peaks corresponding to the W^6+^ state are found at slightly smaller binding energies (35.82 and 37.95 eV). The presence of a satellite peak (W 5p_3/2_) in the W 4f spectra is also observed at 41.59 eV for both as-deposited and annealed films. O 1s spectra of the WO*_x_* films before and after annealing are presented in Figure 3c and Figure 3d, respectively, which have been deconvoluted into three separate peaks. The intense peak at 530.7 eV can be assigned to lattice O atoms (*O*_L_) in the stoichiometric WO_3_ structure, whereas the peaks at 531.5 eV are due to the presence of *O*_V_ in the films [40]. In addition, the presence of surface adsorbates (at 532.6 eV) is observed in both the as-deposited and annealed WO*_x_* films [40]. The results suggest that the relative *O*_V_ concentration in the WO*_x_* films increases from 19 to 25% after vacuum annealing; consequently, a rise in the W^5+^ component is also observed. In contrast, XPS analysis on a 30 nm as-deposited film (Figure S2a,b in Supporting Information File 1) indicates a much higher *O*_V_ concentration (26%) along with an increase in W^5+^ component compared to the as-deposited 6 nm film. Overall, from these results, one can infer that there is an enhanced concentration of *O*_V_ in thicker films in both the as-deposited and annealed case.

**Figure 4:**
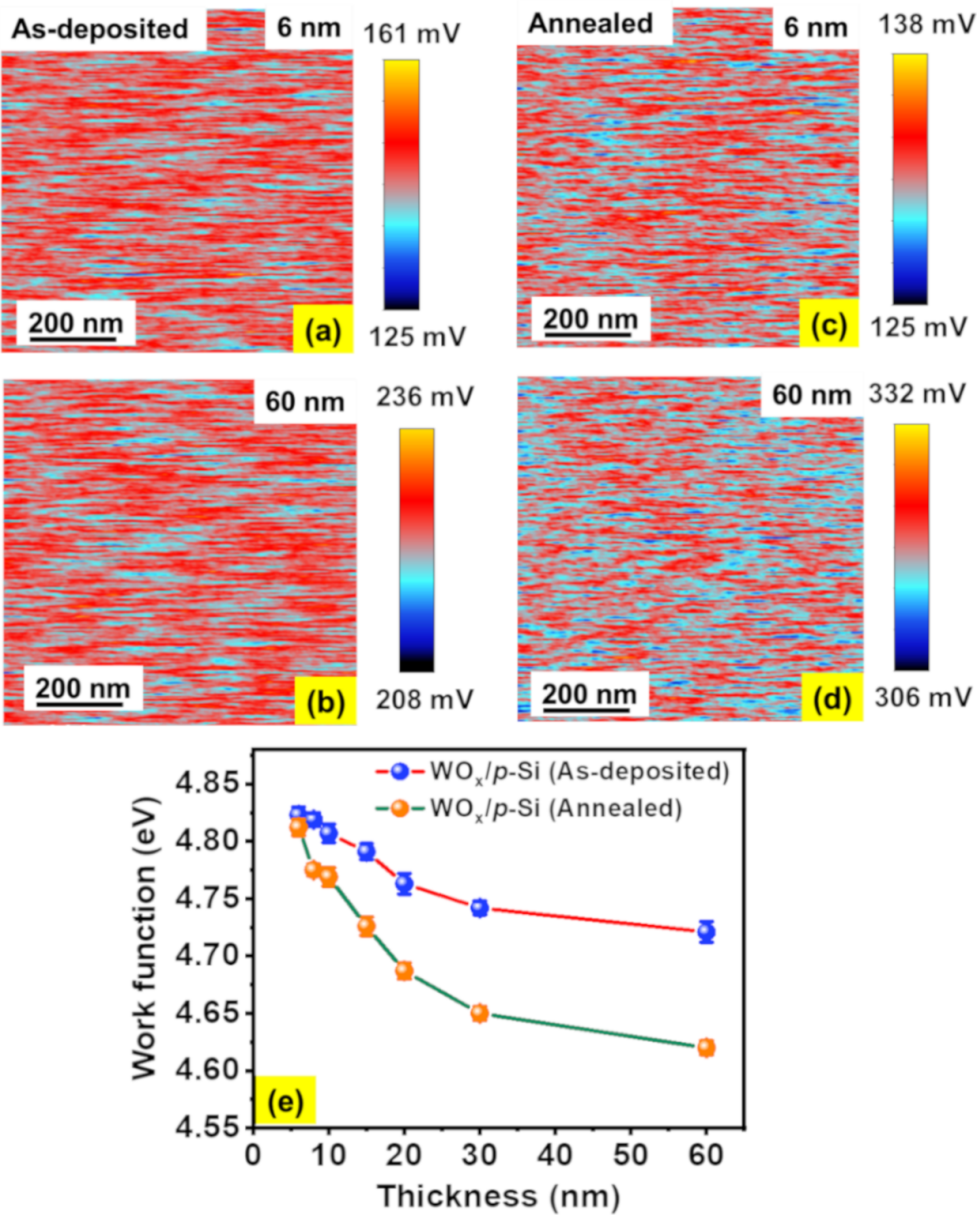
(a, b) and (c, d) *V*_CPD_ maps of, respectively, as-deposited and vacuum annealed WO*_x_* films having thicknesses of 6 and 60 nm. (e) Presents the work function variation with film thickness.

**Figure 5:**
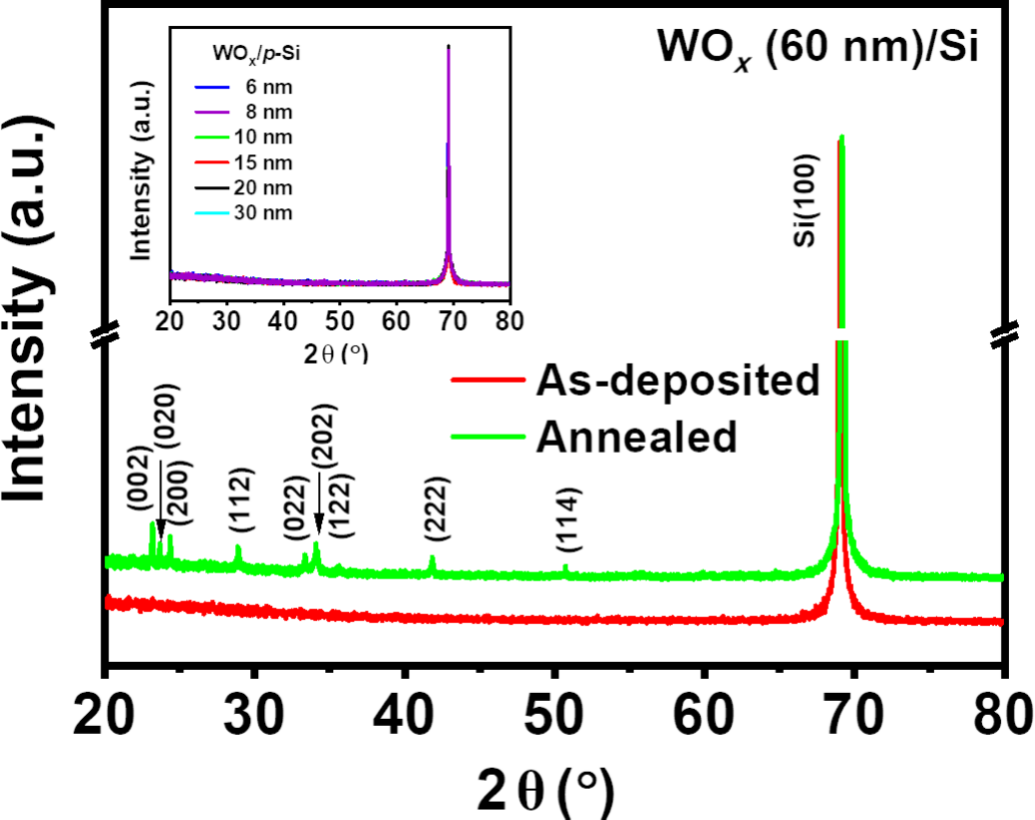
XRD spectra of WO*_x_* films deposited at 87° before and after annealing. The inset shows the XRD spectra of WO*_x_* films having thicknesses varying from 6 to 30 nm.

The variation in *O*_V_ concentration within a metal oxide film is known to influence its bandgap and work function values. As *O*_V_ increases, there is a corresponding rise in electron concentration within the bandgap region, which results in the formation of certain localized electronic states associated with these vacancy defects within the forbidden gap. These states lead to a reduction in bandgap energy and an upward shift of the Fermi level [41–42]. This is the reason behind the observed reduction in bandgap with higher thicknesses (Figure 2b,c) [43]. To probe the Fermi level position, KPFM is a useful tool to acquire valuable information on the work function of a variety of films’ surfaces. Mathematically, the sample work function (ϕ_sample_) can be expressed as:


[2] $$\phi_{\text{sample}}=\phi_{\text{tip}}-eV_{\text{CPD}},$$

where the contact potential difference between the sample and the tip is denoted by *V*_CPD_ and the ϕ_tip_ is the work function of the tip [44]. Figure 4a–d presents the *V*_CPD_ maps of 6 and 60 nm thick films before and after annealing. A comparison between the *V*_CPD_ maps and the respective topographic images in Figure 1 suggest that there is apparently no deviation in *V*_CPD_ among the grain-like topographic features, suggesting uniform *V*_CPD_ variation across the film surface. The work function of the tip was estimated to be 4.94 eV by using highly oriented pyrolytic graphite as a reference, and the work functions of the films were estimated using Equation 2. Figure 4e depicts the variation in work function of NS-WO*_x_* films before and after annealing as a function of the film thickness. It is worth noting that the as-deposited 6 nm film has a maximum work function of 4.82 eV, which gradually decreases to 4.72 eV when the thickness increases to 60 nm. A similar decreasing trend is observed for vacuum-annealed NS-WO*_x_* films, where the work function reduces from 4.81 to 4.62 eV as the film thickness changes from 6 to 60 nm. Another aspect to note from Figure 4e is that the annealed films possess lower work functions than the as-deposited ones, especially for thicker films (60 nm). Apart from having a higher concentration of *O*_V_ in the annealed films, the change in crystallinity after vacuum annealing might also play a role in determining the work function [45]. XRD measurements were carried out on the 60 nm thick film to investigate any possible change in the crystallinity due to vacuum annealing. The XRD data (Figure 5) of a 60 nm-thick NS-WO*_x_* film reveals an amorphous nature of the as-deposited film, which transforms into a polycrystalline monoclinic structure after vacuum annealing. A similar observation is reported in literature where the as-deposited amorphous WO*_x_* films transformed into monoclinic structures after annealing at or above 673 K [2,12,46]. It is to be noted that no apparent peak is visible in annealed films having thicknesses less than or equal to 30 nm (Figure 5, inset). Depending on the material, there can be a critical thickness below which crystallization may not be observed in as-deposited as well as annealed films [33,47–48]. The inhibition of crystallization at lower thicknesses arises mainly because of a considerable contribution of the surface energy to the total Gibbs free energy of crystallization, resulting in a higher crystallization temperature and/or time [47–48]. It can be concluded that the annealing condition (i.e., 673 K, 1 h) used in the present study may not be sufficient to induce crystallinity in the films.

Usually, the work function of polycrystalline WO*_x_* films is reported to vary between 5.7 and 6.7 eV [49]. In the present work, no O_2_ partial pressure was used, so it is expected that all WO*_x_* films are reduced in nature. This explains the observed low work function values of the films, which are mainly due to the presence of a high concentration of *O*_V_ in the same (see XPS results). Greiner et al. have pointed out that a higher work function can be achieved in metal oxides in fully oxidized form compared to their reduced form, and a small change in the stoichiometric ratio is sufficient for a significant change in the material’s work function [42].

**Figure 6:**
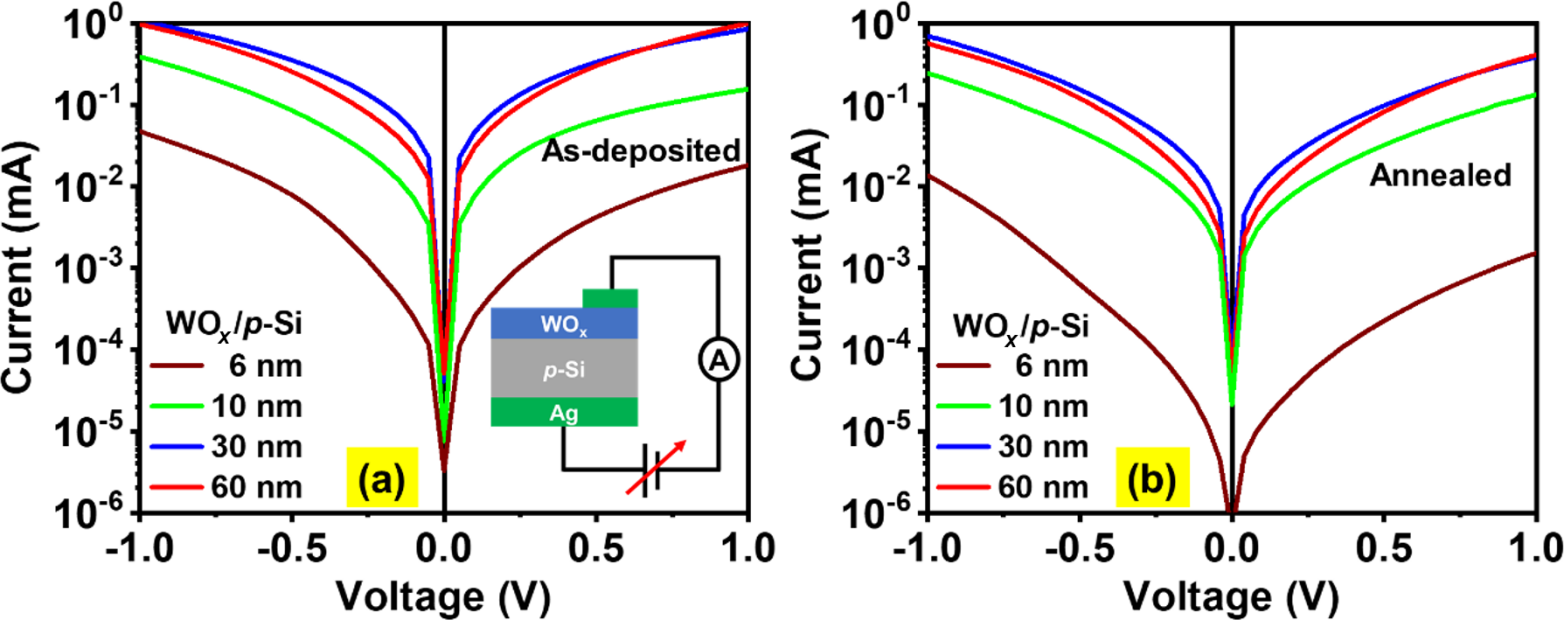
*I*–*V* characteristics of (a) as-deposited and (b) annealed WO*_x_*/p-Si heterostructures for different WO*_x_* film thickness.

Next, a series of NS-WO*_x_*/p-Si heterojunctions was constructed to investigate how the above variation in physicochemical properties influences the current transport through the same. The *I*–*V* characteristics were recorded with a positive bias applied to the p-Si substrate. Figure 6a and Figure 6b show, respectively, the semi-log *I*–*V* curves (linear *I*–*V* curves are presented in Figure S4 of Supporting Information File 1) of WO*_x_*/p-Si heterostructures before and after annealing as a function of the WO*_x_* thickness. A schematic of the measurement configuration is presented in the inset of Figure 6a. An increase in current is apparent as the thickness increases (up to 30 nm). For instance, the current at +1 V increases from 0.1 to 1 mA as the thickness increases from 6 to 30 nm. The XPS results supported by KPFM analysis reveal a higher concentration of *O*_V_ in thicker WO*_x_* films, which mainly act as donors. The presence of a higher amount of *O*_V_ can promote *O*_V_-mediated conduction across the NS-WO*_x_* films [50], as observed in Figure 6.

However, a further increase in thickness to 60 nm saturates the overall current increment due to the dominant effect of series resistance in thicker films (i.e., for 60 nm). Another aspect to note from the *I*–*V* curves is that a drastic rise in current (about one order of magnitude) is seen when the film thickness increases from 6 to 10 nm for both the as-deposited and annealed films (Figure 6a). It may arise from Fermi level de-pinning at the NS-WO*_x_*/p-Si heterojunction when the film thickness increases to 10 nm. To understand this behaviour, in the *I*–*V*-measurement configuration (Figure 6a, inset), the WO*_x_* film can be regarded as an interlayer between the p-Si substrate and the Ag electrode. The glancing angle (87°) growth of a 6 nm film is likely to sustain a large number of metal (Ag)-induced gap states at the NS-WO*_x_*/p-Si interface, leading to Fermi level pinning, the degree of pinning being directly related to film thickness [51]. Evidently, more symmetric *I*–*V* characteristics are observed with increasing film thickness, which can be quantitatively verified from the calculated rectification ratio (RR) values of the NS-WO*_x_*/p-Si heterojunctions, as summarised in Table 1. The RR values are calculated employing Equation 3 for the as-deposited and annealed NS-WO*_x_*/p-Si heterojunctions at three different applied voltages (*V*), viz. ±0.5, ±0.7, and ±1 V, where a higher value of RR indicates a stronger rectifying nature of a junction.


[3] $$\text{RR}\left(V\right)=\frac{I\left(-V\right)}{I\left(V\right)}$$

As can be seen from Table 1, an increase in the film thickness minimizes the RR value of the junction, thereby leading to more symmetric *I*–*V* curves. Nevertheless, one can infer from the observed low values of RR (Table 1) that all the as-deposited NS-WO*_x_*/p-Si samples show a quasi-ohmic conduction behaviour, which implies that tunnelling may be responsible for the current transport. The post-growth annealing in vacuum of NS-WO*_x_*/p-Si samples generates an enhanced *O*_V_ concentration (as observed from the XPS analysis) in the WO*_x_* films, which eventually contributes to a higher number of gap states within the film as well as at the WO*_x_*/p-Si interface. Since the Fermi level pinning becomes stronger with the increasing number of interface gap states [52–53], this explains the comparatively higher values of RR in all annealed samples (Table 1). Thus, there exists a critical thickness (30 nm) of the NS-WO*_x_* layers in terms of effective elimination of Fermi level pinning at the interface and increased series resistance in the film.

**Table 1:** Rectification ratio values (RR) for as-deposited and annealed WO*_x_* films with various thicknesses.

Sample	Thickness (nm)	RR		

		±0.5 V	±0.7 V	±1.0 V

WO*_x_*/p-Si (as-deposited)	6	1.82	2.04	2.59
	10	1.30	1.74	2.49
	30	1.02	1.08	1.22
	60	0.81	0.91	0.96

WO*_x_*/p-Si (annealed)	6	2.76	4.70	9.04
	10	1.40	1.58	1.88
	30	1.48	1.66	1.77
	60	1.37	1.38	1.37

Describing the *I*–*V* characteristics of the heterojunction, it can be noted that no correlation can be drawn between the surface work function values obtained from KPFM analysis and the resulting band offsets at the hetero-interface [54–55]. However, the investigation on the variation in work function with thickness for GLAD-grown WO*_x_* films in this study is valuable towards potential device applications, where work function optimization among consecutive layers is imperative. Moreover, the adopted rf sputtering technique in the present work ensures reduced surface damage of the underlying Si substrate, which is otherwise prominent in case of DC sputtering, commonly adopted for WO*_x_* growth under GLAD configuration. For a better assessment of the present work and existing literature, a table of comparison (Table S1) is provided in Supporting Information File 1.

Overall, the detailed study exploring a wide range of tunability regarding electrical and optical properties in WO*_x_* films as functions of the thickness points towards its importance for various device applications including electrochromic devices, RRAM, and LEDs, and even in designing carrier-selective contacts for solar cells.

## Conclusion

A series of glancing angle-deposited NS-WO*_x_* thin films (6–60 nm) on p-Si substrates are investigated to achieve insights into their tuneable structural, optical, and electrical properties, such as crystallinity, bandgap, work function, and diode characteristics. As revealed from the XRD studies, as-deposited NS-WO*_x_* films are amorphous in nature, whereas post-growth vacuum-annealed (at 673 K for 1 h) films show an amorphous-to-crystalline structural phase transition. XPS analysis confirms an increasing concentration of defect density in the form of oxygen vacancies with increasing film thickness and also due to the post-growth annealing, which corroborates well the KPFM and UV–vis–NIR spectrophotometric analyses showing the reduction in work function and bandgap values. *I*–*V* characteristics of WO*_x_*/p-Si heterojunctions reveal a nearly symmetric nature in the as-deposited films, compared to the annealed ones, indicating a quasi-ohmic nature of the junction. Increased rectification ratios are observed for thinner WO*_x_* films, which are insufficient to overcome the metal electrode-induced gap states at the interface leading to Fermi level pinning. Improved current conduction across the heterojunction with increasing film thickness indicates a possible role of oxygen vacancies in facilitating smooth charge transport till the thickness reaches 30 nm, above which the series resistance effect within the WO*_x_* film starts to dominate. Overall, this study demonstrates a wide range of tunability in structural, optical, and electrical properties of NS-WO*_x_* thin films through controlling microstructure and film thickness. This will be useful for optoelectronic applications in photovoltaics where such films are used as a carrier-selective contact.

## Supporting Information

Cross-sectional SEM images of 60 and 120 nm WO*_x_* films, XPS spectra of 30 nm WO*_x_* film, Tauc plots of as-deposited WO*_x_* films, and linear *I*–*V* curves of as-deposited and annealed WO*_x_* films.

File 1 Supplementary data.

## Data Availability

The data that supports the findings of this study is available from the corresponding author upon reasonable request.

## References

[1] Yao Y, Sang D, Zou L, Wang Q, Liu C (2021). Nanomaterials.

[2] Zheng H, Ou J Z, Strano M S, Kaner R B, Mitchell A, Kalantar‐zadeh K (2011). Adv Funct Mater.

[3] Cook B, Liu Q, Butler J, Smith K, Shi K, Ewing D, Casper M, Stramel A, Elliot A, Wu J (2018). ACS Appl Mater Interfaces.

[4] Niklasson G A, Granqvist C G (2007). J Mater Chem.

[5] Cheng C-P, Kuo Y, Chou C-P, Cheng C-H, Teng T P (2014). Appl Phys A: Mater Sci Process.

[6] Georg A, Georg A, Graf W, Wittwer V (2008). Vacuum.

[7] Fortunato J, Zydlewski B Z, Lei M, Holzapfel N P, Chagnot M, Mitchell J B, Lu H-C, Jiang D-e, Milliron D J, Augustyn V (2023). ACS Photonics.

[8] Yu B, Wang J, Mo X, Yang X, Wang W, Cai X (2020). Colloids Surf, A.

[9] Shinde N M, Jagadale A D, Kumbhar V S, Rana T R, Kim J, Lokhande C D (2015). Korean J Chem Eng.

[10] Kostis I, Vasilopoulou M, Papadimitropoulos G, Stathopoulos N, Savaidis S, Davazoglou D (2013). Surf Coat Technol.

[11] Yuan Y, Zhang X, Li D, Zhang X, Wang L, Lu Z, Liu L, Chi F (2020). Phys Chem Chem Phys.

[12] Lee C-Y, Aziz M I A, Wenham S, Hoex B (2017). Jpn J Appl Phys.

[13] Nasser H, Gülnahar M, Mehmood H, Canar H H (2022). SSRN Electron J.

[14] Wei S, Ma Y, Chen Y, Liu L, Liu Y, Shao Z (2011). J Hazard Mater.

[15] Zhang J, Lu H, Liu C, Chen C, Xin X (2017). RSC Adv.

[16] Li N, Fu S, Wu J, Li X, Zhou J, Wang Y, Zhang X, Liu Y (2020). Appl Phys Lett.

[17] Wang L, Cheng S, Wu C, Pei K, Song Y, Li H, Wang Q, Sang D (2017). Appl Phys Lett.

[18] Yin C, Zhu S, Zhang D (2017). RSC Adv.

[19] Bivour M, Zähringer F, Ndione P, Hermle M (2017). Energy Procedia.

[20] Mahjabin S, Hossain M I, Haque M M, Bashar M S, Jamal M S, Shahiduzzaman M, Muhammad G, Sopian K, Akhtaruzzaman M (2022). Appl Phys A: Mater Sci Process.

[21] Xu X, Arab Pour Yazdi M, Rauch J-Y, Salut R, Billard A, Potin V, Martin N (2015). Mater Today: Proc.

[22] Huang Z-F, Song J, Pan L, Zhang X, Wang L, Zou J-J (2015). Adv Mater (Weinheim, Ger).

[23] Calixto-Rodriguez M, García H M, Nair M T S, Nair P K (2013). ECS J Solid State Sci Technol.

[24] Pang S, Gong L, Du N, Luo H, Yu K, Gao J, Zheng Z, Zhou B (2019). Mater Today Energy.

[25] Zheng M, Tang H, Hu Q, Zheng S, Li L, Xu J, Pang H (2018). Adv Funct Mater.

[26] Rydosz A, Dyndał K, Kollbek K, Andrysiewicz W, Sitarz M, Marszałek K (2020). Vacuum.

[27] Kumar M, Som T (2015). Nanotechnology.

[28] Singh R, Sivakumar R, Srivastava S K, Som T (2020). Appl Surf Sci.

[29] Kumar M, Singh R, Nandy S, Ghosh A, Rath S, Som T (2016). J Appl Phys.

[30] Chatterjee S, Kumar M, Gohil S, Som T (2014). Thin Solid Films.

[31] Hawkeye M M, Taschuk M T, Brett M J (2014). Glancing Angle Deposition of Thin Films.

[32] Kumar M, Kanjilal A, Som T (2013). AIP Adv.

[33] Singh R, Sivakumar R, Kumar Srivastava S, Som T (2021). Appl Surf Sci.

[34] Dutta A, Singh R, Srivastava S K, Som T (2019). Sol Energy.

[35] Aly S A, Akl A A (2015). Chalcogenide Lett.

[36] Charles C, Martin N, Devel M, Ollitrault J, Billard A (2013). Thin Solid Films.

[37] Kwong W L, Savvides N, Sorrell C C (2012). Electrochim Acta.

[38] Weinhardt L, Blum M, Bär M, Heske C, Cole B, Marsen B, Miller E L (2008). J Phys Chem C.

[39] Ji R, Zheng D, Zhou C, Cheng J, Yu J, Li L (2017). Materials.

[40] Bourdin M, Mjejri I, Rougier A, Labrugère C, Cardinal T, Messaddeq Y, Gaudon M (2020). J Alloys Compd.

[41] El-Nahass M M, Soliman H S, El-Denglawey A (2016). Appl Phys A: Mater Sci Process.

[42] Greiner M T, Chai L, Helander M G, Tang W-M, Lu Z-H (2012). Adv Funct Mater.

[43] Malliga P, Pandiarajan J, Prithivikumaran N, Neyvasagam K (2014). IOSR J Appl Phys.

[44] Kumar M, Singh R, Som T (2018). Appl Surf Sci.

[45] Irfan I, James Turinske A, Bao Z, Gao Y (2012). Appl Phys Lett.

[46] Srichaiyaperk T, Aiempanakit K, Horprathum M, Eiamchai P, Chananonnawathorn C, Limwichean S, Chindaudom P (2014). Adv Mater Res.

[47] Nie X, Ma F, Ma D, Xu K (2015). J Vac Sci Technol, A.

[48] Zhang L, Zhang J, Jiao H, Bao G, Wang Z, Cheng X (2017). Thin Solid Films.

[49] Mews M, Korte L, Rech B (2016). Sol Energy Mater Sol Cells.

[50] Li J-J, Zhang M, Weng B, Chen X, Chen J, Jia H-P (2020). Appl Surf Sci.

[51] Zhang Y, Han G, Wu H, Wang X, Liu Y, Zhang J, Liu H, Zheng H, Chen X, Liu C (2018). Nanoscale Res Lett.

[52] Bampoulis P, van Bremen R, Yao Q, Poelsema B, Zandvliet H J W, Sotthewes K (2017). ACS Appl Mater Interfaces.

[53] Cowley A M, Sze S M (1965). J Appl Phys.

[54] Song X M, Huang Z G, Gao M, Chen D Y, Fan Z, Ma Z Q (2021). Int J Photoenergy.

[55] Gerling L G, Voz C, Alcubilla R, Puigdollers J (2017). J Mater Res.

