# Supplementary material for: Controllable physicochemical properties of WOx thin films grown under glancing angle
Source: Beilstein J Nanotechnol. 2024 Apr 2;15:350–9. doi: 10.3762/bjnano.15.31 (PMC10999991; doi:10.3762/bjnano.15.31)
Supplement: File 1 — Supplementary data. [file Beilstein_J_Nanotechnol-15-350-s001.pdf]

BEILSTEIN JOURNAL OF NANOTECHNOLOGY

Supporting Information

for

Controllable physicochemical properties of WO_x thin films grown under glancing angle

Rupam Mandal, Aparajita Mandal, Alapan Dutta, Rengasamy Sivakumar, Sanjeev Kumar Srivastava and Tapobrata Som

Beilstein J. Nanotechnol. **2024,** *15,* 350–359. doi:10.3762/bjnano.15.31

Supplementary data

License and Terms: This is a supporting information file under the terms of the Creative Commons Attribution License (https://creativecommons.org/licenses/by/4.0). Please note that the reuse, redistribution and reproduction in particular requires that the author(s) and source are credited and that individual graphics may be subject to special legal provisions.
The license is subject to the *Beilstein Journal of Nanotechnology* terms and conditions: (https://www.beilstein-journals.org/bjnano/terms)

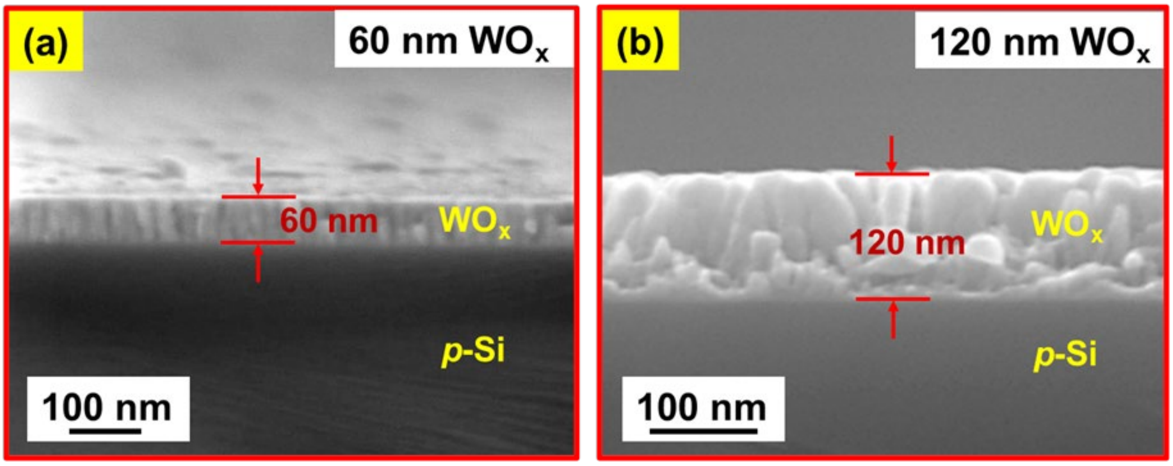

Figure S1: (a, b) XSEM images of 60 and 120 nm thick WO_x thin films deposited on p-Si substrates.

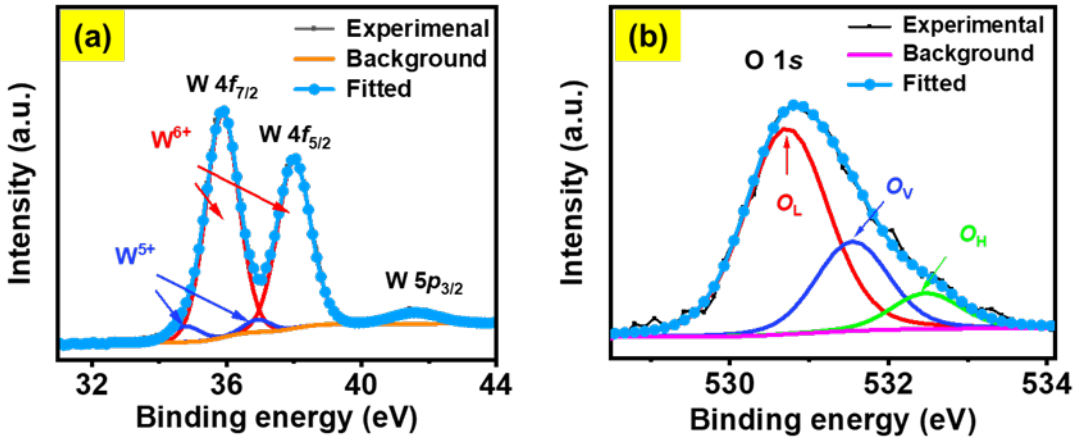

Figure S2: (a, b) XPS W 4f and O 1s core level spectra of as-deposited WO_x films having a thickness of 30 nm.

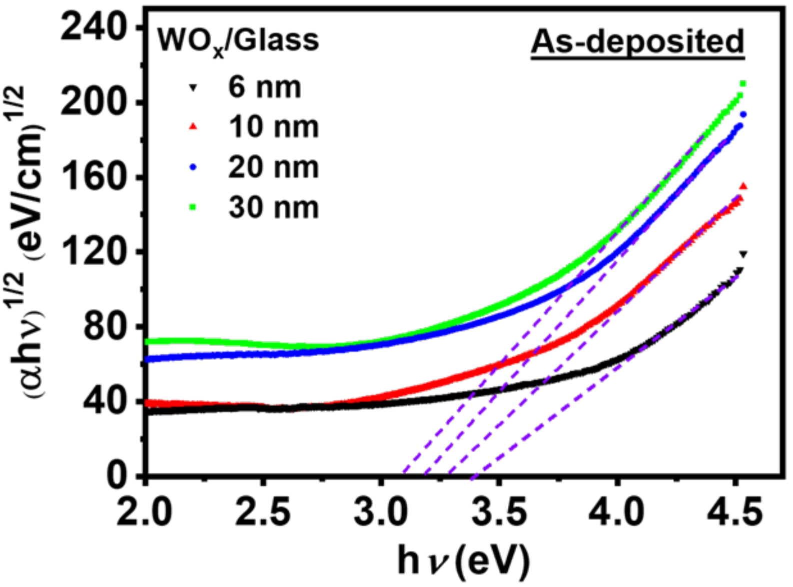

Figure S3: Tauc plots of the as-deposited nanostructured WO_x for different film thicknesses. The dashed lines indicate the intercept of the extrapolates on the x axis, providing the corresponding bandgap energies.

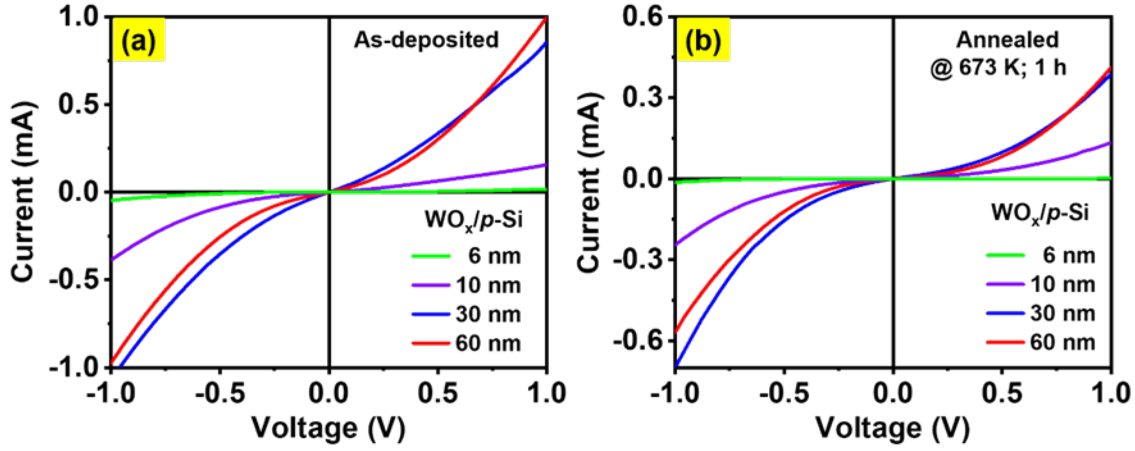

Figure S4: Linear I–V curves of (a) as-deposited and (b) annealed WO_x/p-Si heterostructures for different WO_x film thicknesses.

Table S1: Comparison between the present work and previously reported works on nanostructured WO_3 films.

**Film thickness (nm)**	**Growth technique**	**Target used**	**Growth geometry**	**Optical studies**	**Electrical studies**	**Local probe studies**	**Post-growth annealing**	**Ref.**
25–54	DC sputtering	W	GLAD	transmittance, bandgap, refractive index	*I–V*	NA	400 °C	[1]
500	reactive DC magnetron sputtering	W	GLAD	NA	current response for gas sensing	NA	400 and 500 °C	[2]
100	pulsed DC magnetron sputtering	W	GLAD	none (only structural and morphological studies)	NA	NA	500 °C	[3]
270, 500	e-beam evaporation	WO_3	GLAD	transmittance	*I–V*	NA	100, 200, 300, 350, and 400 °C	[4]
1000	reactive DC magnetron sputtering	W	GLAD	transmittance, refractive index, bandgap	NA	NA	NA	[5]
300	reactive DC magnetron sputtering	W	GLAD	transmittance	NA	NA	NA	[6]
6–120 nm	RF magnetron sputtering	WO_3	GLAD	transmittance, bandgap	*I–V*	Local work function	400 °C	present work

References

1. Rydosz, R.; Dyndał, K.; Kollbe, K.; Andrysiewicz, W.; Sitarz, M.; Marszałek, K. *Vacuum* **2020,** *177,* 109378. doi:10.1016/j.vacuum.2020.109378
2. Horprathum, M.; Frankel, D. J.; Lad, R. J. *Sens. Actuators, B* **2013,** *176,* 685–691. doi:10.1016/j.snb.2012.09.077
3. Deniz, D.; Frankel, D. J.; Lad, R. J. *Thin Solid Films* **2010,** *518,* 4095–4099. doi:10.1016/j.tsf.2009.10.153
4. Yuan, J.; Wang, B.; Wang, H.; Chai, Y.; Jin, Y.; Qi, H.; Shao, H. *Appl. Surf. Sci.* **2018,** *447,* 471–478. doi:10.1016/j.apsusc.2018.03.248
5. Sakkas, C.; Rauch, J.-Y.; Cote, J.-M.; Tissot, V.; Gavoille, J.; Martin, N. *Coatings* **2021,** *11,* 438. doi:10.3390/coatings11040438
6. Salawan, C.; Aiempanakit, M.; Aiempanakit, K.; Chananonnawathorn, C.; Eiamchai, P.; Horprathum, M. *Mater. Today: Proc.* **2017,** *4,* 6423–6429. doi:10.1016/j.matpr.2017.06.148
